# Supplementary material for: Reorganization of structural connectivity in the brain supports preservation of cognitive ability in healthy aging
Source: Netw Neurosci. 2024 Oct 1;8(3):837–59. doi: 10.1162/netn_a_00377 (PMC11398719; doi:10.1162/netn_a_00377)
Supplement: Supplementary file 1 [file netn-8-3-837-s001.pdf]

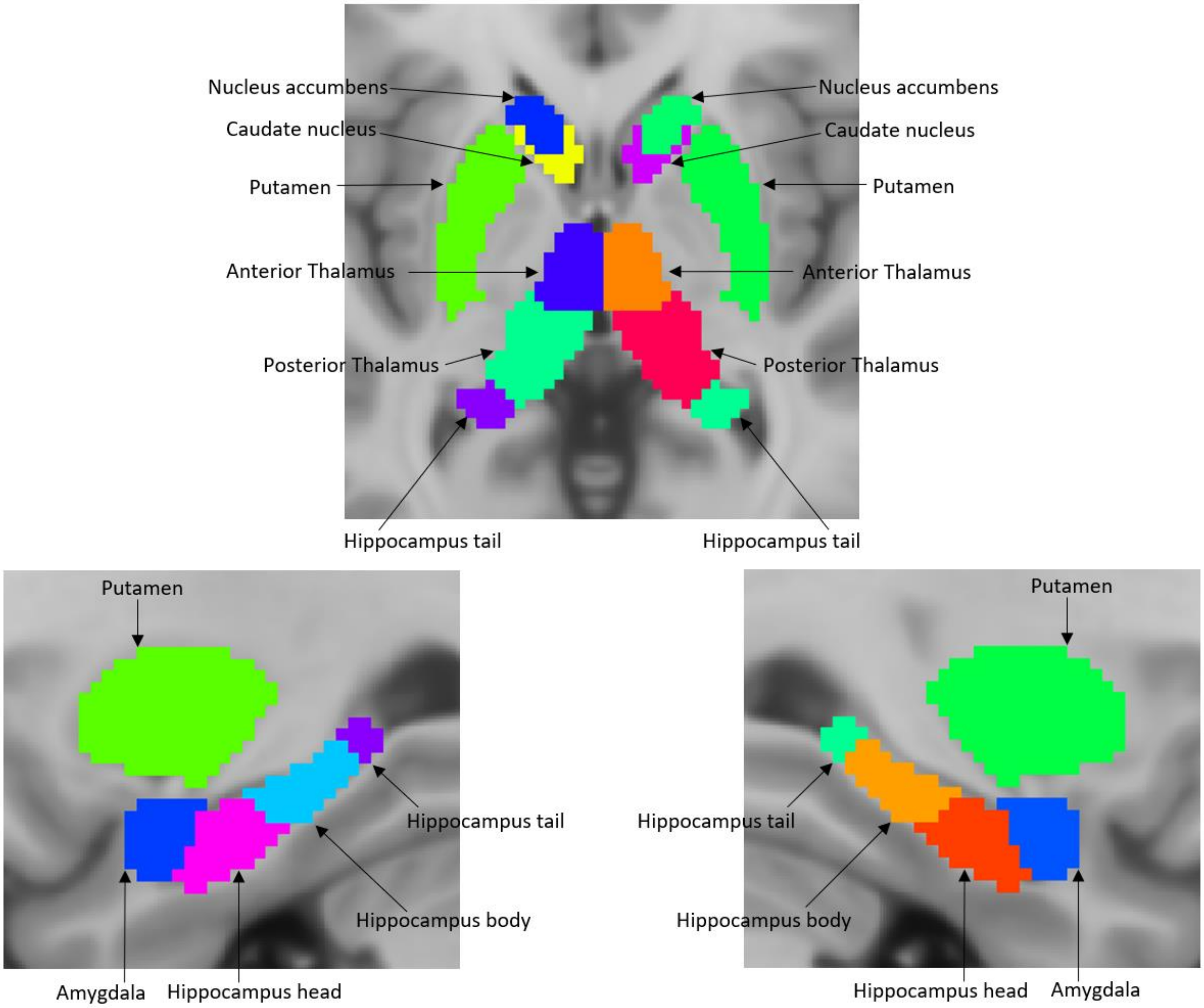

Supplementary Figure 1. Tian et al. (2020) subcortical parcellation labels.

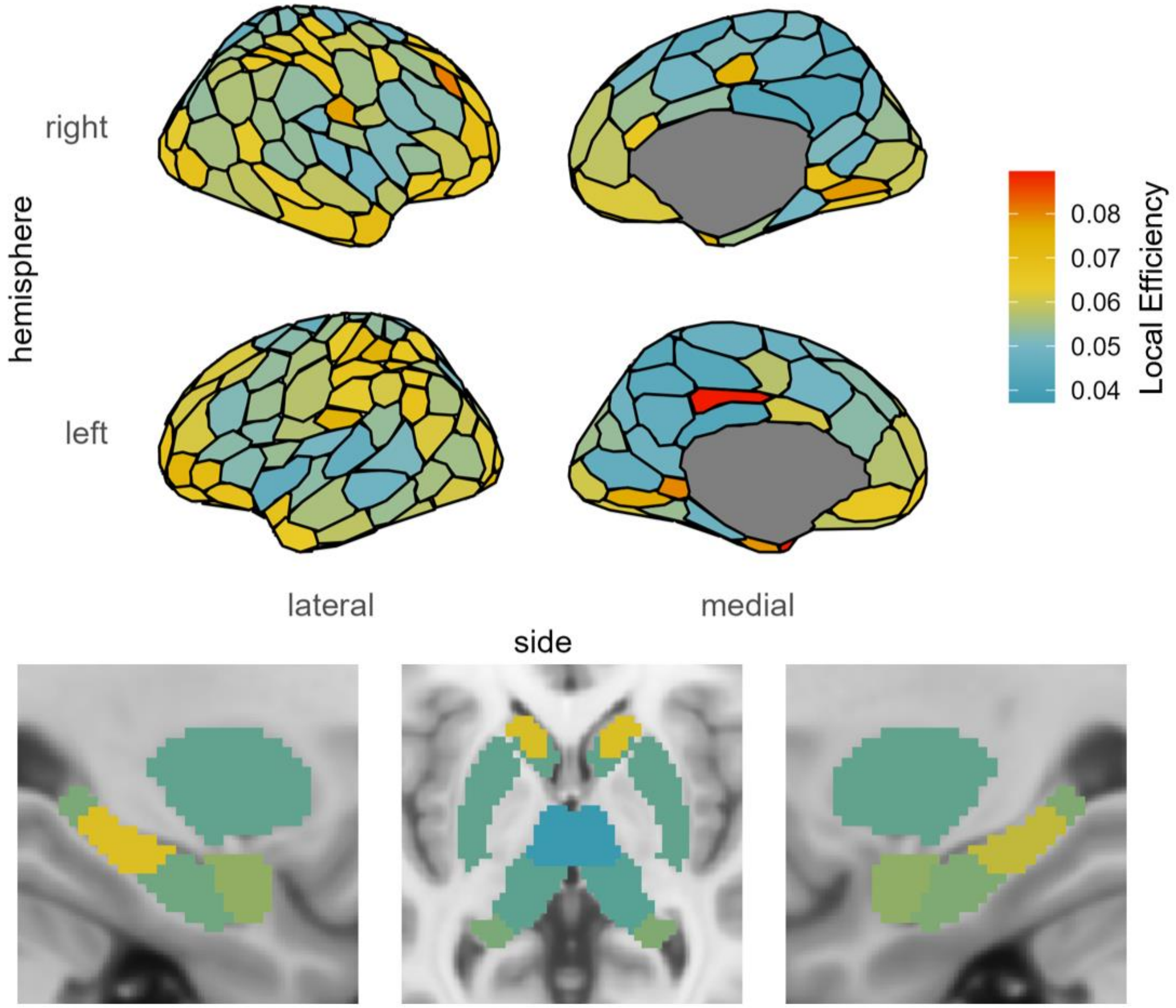

Supplementary Figure 2. Brain map of mean local efficiency for all participants.
